# Genomic characterization of carbapenem-non-susceptible *Pseudomonas aeruginosa* in Singapore

**DOI:** 10.1080/22221751.2021.1968318

**Published:** 2021-08-31

**Authors:** Jocelyn Qi-Min Teo, Cheng Yee Tang, Jie Chong Lim, Shannon Jing-Yi Lee, Si Hui Tan, Tse-Hsien Koh, James Heng-Chiak Sim, Thuan-Tong Tan, Andrea Lay-Hoon Kwa, Rick Twee-Hee Ong

**Affiliations:** a Department of Pharmacy, Singapore General Hospital, Singapore, Singapore; b Saw Swee Hock School of Public Health, National University of Singapore and National University Health System, Singapore, Singapore; c Department of Pharmacy, National University of Singapore, Singapore, Singapore; d Department of Microbiology, Singapore General Hospital, Singapore, Singapore; e Department of Infectious Diseases, Singapore General Hospital, Singapore, Singapore; f Singhealth Duke-NUS Medicine Academic Clinical Programme, Singapore, Singapore; g Emerging Infectious Diseases, Duke-National University of Singapore Medical School, Singapore, Singapore

**Keywords:** Pseudomonas aeruginosa, whole-genome sequencing, resistome, clones, multi-drug resistant, genomic surveillance

## Abstract

*Pseudomonas aeruginosa* is a clinically important pathogen implicated in many hospital-acquired infections. Its propensity to acquire broad-spectrum resistance has earned the organism its status as a severe public health threat requiring urgent control measures. While whole-genome sequencing-based genomic surveillance provides a means to track antimicrobial resistance, its use in molecular epidemiological surveys of *P. aeruginosa* remains limited, especially in the Southeast Asian region. We sequenced the whole genomes of 222 carbapenem-non-susceptible *P. aeruginosa* (CNPA) isolates collected in 2006–2020 at the largest public acute care hospital in Singapore. Antimicrobial susceptibilities were determined using broth microdilution. Clonal relatedness, multi-locus sequence types, and antimicrobial resistance determinants (acquired and chromosomal) were determined. In this study, CNPA exhibited broad-spectrum resistance (87.8% multi-drug resistance), retaining susceptibility only to polymyxin B (95.0%) and amikacin (55.0%). Carbapenemases were detected in 51.4% of the isolates, where IMP and NDM metallo-*β*-lactamases were the most frequent. Carbapenem resistance was also likely associated with OprD alterations or efflux mechanisms (ArmZ/NalD mutations), which occurred in strains with or without carbapenemases. The population of CNPA in the hospital was diverse; the 222 isolates grouped into 68 sequence types (ST), which included various high-risk clones. We detected an emerging clone, the NDM-1-producing ST308, in addition to the global high-risk ST235 clone which was the predominant clone in our population. Our results thus provide a “snapshot” of the circulating lineages of CNPA locally and the prevailing genetic mechanisms contributing to carbapenem resistance. This database also serves as the baseline for future prospective surveillance studies.

## Introduction

*Pseudomonas aeruginosa* are Gram-negative bacteria responsible for various nosocomial infections, including those of the blood, lungs, and urinary tract [1,2]. Difficult-to-treat antibiotic-resistant *P. aeruginosa* is becoming increasingly common as the already intrinsically resistant organism develops resistance to the broad-spectrum carbapenems [3]. Even with the advent of newer agents, carbapenem-non-susceptible *P. aeruginosa* (CNPA) infection remains one of the most challenging infections to treat, contributing to excess 30-day mortality in patients [4]. Patients with CNPA infections have also incurred longer and costlier hospitalizations than those infected with susceptible strains [5]. As a result, the World Health Organization has classified CNPA as critical priority pathogens for the development of new effective antibiotics, underscoring the dangers these organisms pose to public health [6].

Carbapenem resistance in *P. aeruginosa* is often multifactorial and emerges when driven by selection pressure from continued antibiotic use [7]. Mechanisms mediating resistance are often mutational in nature, leading to the alteration or a loss of outer membrane porins, upregulation of efflux pumps, or hyperproduction of chromosomal AmpC *β*-lactamase [8]. Resistance may also result from the acquisition of carbapenemases on mobile elements. This diverse range of resistance genes can further spread amongst CNPA via transformation, transduction, and/or conjugation. As a result, the combination of various resistance mechanisms may produce a spectrum of isolates with varying genotypes and hence varying phenotypes [9].

Owing to a propensity of ever-changing genotypes and phenotypes in CNPA, continuous epidemiological surveillance of antimicrobial-resistant pathogens like CNPA is therefore essential to inform infection control and treatment practices [10]. Previous studies have largely focused on specific outbreaks of carbapenemase-producing strains, hence the molecular epidemiology of CNPA in Singapore remains largely unknown [11–13]. The advent of high-throughput sequencing has facilitated the in-depth genomic characterization of pathogens, given its superior resolution and reproducibility compared to traditional DNA band-based methods. Whole-genome sequencing (WGS) is able to reveal insights into the dissemination of high-risk clones and underpinnings of antibiotic resistance, potentially leading to targeted and effective control of these pathogens. In this study, we conducted a retrospective WGS survey including isolates collected over 15 years in order to describe the antimicrobial susceptibilities, molecular epidemiology, and prevalence rate of carbapenemase production amongst CNPA in Singapore.

## Methods

### Study setting

This study is conducted at the Singapore General Hospital (SGH), the largest public acute care tertiary hospital in Singapore. The hospital has approximately 1800 beds and accounts for approximately 25% of the total acute hospital beds in the public sector and 20% of acute beds nationwide. A wide range of medical and surgical specialties are offered by the hospital and the centre is the national/regional referral centre for services such as plastic surgery and burns, renal medicine, nuclear medicine, pathology, and haematology.

### Bacterial isolates

A total of 222 non-duplicate clinical CNPA isolates from various clinical sites, which exhibited non-susceptibility to at least one carbapenem (doripenem, meropenem, imipenem) were included in this survey (Table S1) [14]. These isolates were collected at SGH’s Pharmacy Research Laboratory between 2006 and 2020 as part of an informal surveillance study of carbapenem-non-susceptible gram-negative organisms, including *P. aeruginosa* [15].

All isolates were identified using VITEK® GNI+ cards with the VITEK® 2 instrument (bioMérieux, Hazelwood, MO, USA) and/or matrix-assisted laser desorption/ionization time-of-flight mass spectrometry (MALDI-TOF MS) system (Bruker Daltonik, Germany) as part of the institution’s microbiology laboratory routine workflow.

### Antibiotic susceptibilities

Antimicrobial susceptibilities of the following antibiotic categories were tested: (1) carbapenems (imipenem, meropenem, doripenem), (2) extended-spectrum cephalosporins (cefepime), (3) aztreonam, (4) piperacillin/tazobactam, (5) aminoglycosides (amikacin, gentamicin), (6) fluoroquinolone (levofloxacin), (7) polymyxin B, and (8) new agents (ceftazidime-avibactam, ceftolozane-tazobactam). Minimum inhibitory concentrations (MICs) were obtained using customized 96-well microbroth dilution panels (TREK Diagnostics, East Grinstead, UK) in accordance with the manufacturer’s recommendations and interpreted according to Clinical & Laboratory Standards Institute (CLSI) breakpoints [16]. *P. aeruginosa* ATCC 27853 was used as the quality control strain.

CNPA isolates were defined as multi-drug resistant (MDR) if they were also non-susceptible to at least one agent in ≥ 2 antibiotic categories (extended-spectrum cephalosporins, fluoroquinolones, aminoglycosides, piperacillin-tazobactam) other than carbapenems [14]. Additionally, isolates were defined as difficult-to-treat resistant (DTR) phenotypes, i.e. having treatment-limiting resistance to all first-line agents, if they were non-susceptible to all of the following agents: imipenem, meropenem, doripenem, cefepime, piperacillin-tazobactam, aztreonam, and levofloxacin [3].

### DNA preparation and whole genome sequencing

Overnight bacterial cultures in cation-adjusted Muller-Hinton were prepared and used for genomic DNA extraction using the DNeasy® Blood & Tissue Kit (QIAGEN GmbH, Hilden, Germany) as per the manufacturer’s instructions. The genomic DNA was then sent for paired-end WGS using MiSeq/HiSeq systems (Illumina Inc., CA, USA), with a resultant sequencing depth of at least 100-fold. Raw sequences were assessed for quality using FastQC (v0.11.3, Babraham Institute), followed by removal of adaptors and poor-quality bases and sequences using Trimmomatic [17,18]. Trimmed sequences were then assembled *de novo* using the SPAdes software [19].

### Antimicrobial resistance profiling, serotyping, and virulence gene characterization

Acquired resistance genes were identified using the NCBI-AMRFinder database [20]. Selected chromosomal gene targets associated with antibiotic resistance were analysed by aligning the trimmed reads to the PAO1 reference genome (GenBank: AE004091.2), and variants were called using Snippy (v4.6.0) (available at https://github.com/tseemann/snippy). The gene targets analysed included those in the quinolone-resistance-determining regions (QRDRs) (*gyrA*, *gyrB, parC*, *parE*); genes mediating *β*-lactam resistance such as (*ampC*, *ampD*, *ampDh2*, *ampDh3*, *ampR*, *dacB*, *ftsI*); genes related to polymyxin resistance (*colR*, *colS*, *cprR*, *cprS*, *pmrA*, *pmrB*, *phoP*, *phoQ*); the gene encoding fosfomycin resistance (*glpT*), those encoding and regulating the efflux systems (*mexR*, *mexZ*, *mexS*, *mexT*, *nfxB*, *nalC*, *nalD*, *armZ*, *parS*, *parR*); and the *oprD* porin gene. Only non-synonymous amino acid substitutions which were identified as deleterious by the Protein Variation Effect Analyzer (PROVEAN) software tool (http:// provean.jcvi.org/index.php) were reported [21]. Plasmids were identified using PlasmidFinder and PlasmidSeeker [22,23].

*In silico* serotyping of the isolates was performed using the *P. aeruginosa* serotypes (PAst) script available on https://github.com/Sandramses/PAst [24]. To detect the presence of the *exo* virulence genes, we performed Basic Local Alignment Search Tool (BLAST) searches to find the orthologous sequences for *exo*S (PAO1 strain: accession NC_002516.2) and *exo*U (PA103 strain: accession U97065.1) in our isolates.

### *In silico* multi-locus sequence typing and phylogenetic analyses

Sequence types (STs) were identified by BLAST using the scheme in the PubMLST database (https://pubmlst.org/paeruginosa/). To construct the phylogenetic tree, the single nucleotide polymorphisms (SNPs) identified with Snippy were used to generate a core SNP alignment. Recombinant regions identified by Gubbins (v2.4.1) were removed from the core SNP alignment prior to tree construction [25]. The maximum likelihood tree was inferred with IQ-TREE (v2.0.3) using the GTR + F+I + G4 nucleotide substitution model, which was the best model selected by ModelFinder, and visualized using a modified R script from https://github.com/katholt/plotTree [26,27].

## Results

### Antimicrobial susceptibility

The antimicrobial susceptibility profiles of CNPA are summarized in Table 1. The isolates exhibited high levels of non-susceptibility to most antibiotics tested. A total of 195 (87.8%) were classified as MDR phenotypes, of which 155 of these isolates were also DTR. Twenty-seven isolates (12.2%) were neither MDR nor DTR, all of which were non-carbapenemase-producing. Susceptibility rates for most *β*-lactams were < 15%. We also observed CNPA isolates (47/222, 21.2%) which remained susceptible to either aztreonam and/or cefepime, and/or piperacillin-tazobactam, suggesting that local CNPA are not necessarily resistant to extended-spectrum cephalosporins.

**Table 1.** Antimicrobial susceptibility patterns of 222 CNPA isolates.

Antibiotic	S (%)	I/SDD (%)	R (%)	MIC data (mg L^−1^)		
				MIC_50_	MIC_90_	Range
Doripenem	12.2	11.2	76.6	32	≥ 64	≤ 0.25–≥ 64
Imipenem	2.7	7.2	90.1	32	≥ 64	2–≥ 64
Meropenem	8.1	7.7	84.2	≥ 64	≥ 64	≤ 0.25–≥ 64
Aztreonam	12.6	28.8	58.6	32	≥1 28	4–≥ 128
Cefepime	15.8	9.9	74.3	≥ 128	≥ 128	≤ 1–≥ 128
Piperacillin/tazobactam	11.8	8.6	79.6	≥ 256/4	≥ 256/4	4/4–≥ 256/4
Levofloxacin	14.4	7.7	77.9	32	≥ 64	≤ 0.25–64
Amikacin	55.0	6.7	38.3	16	≥ 128	≤ 1–≥ 128
Gentamicin	30.6	2.7	66.7	–	–	–
Polymyxin B	–	95.0	5.0	1	2	≤ 0.25–4
Ceftazidime/avibactam^a^	67.0	–	33.0	4	≥ 128/4	1–≥ 128/4
Ceftolozane/tazobactam^a^	64.2	9.2	26.6	2	≥ 128/4	1–≥ 128/4

Notes: I, intermediate; S, susceptible; SDD, susceptible dose-dependent; R, resistant.

^a^ Only assessed for 109 non-metallo-*β*-lactamases producing CNPA isolates (inclusive of 108 carbapenemase-negative + 1 KPC-producing isolates).

Levofloxacin susceptibility (14.4%) was poor, while CNPA exhibited moderate susceptibility (55%) to amikacin. Polymyxin resistance was rare (5.0%), although majority (206/222, 92.8%) of the isolates were only one dilution or at the intermediate breakpoint of 2 mg L^−1^. In non-metallo-*β*-lactamases producers, the susceptibility rates of novel β-lactamase/β-lactamase inhibitors, ceftolozane-tazobactam, and ceftazidime-avibactam were similar (64.2% and 67.0% respectively).

### Molecular resistance characterization

#### Acquired resistome

**Figure 1.**
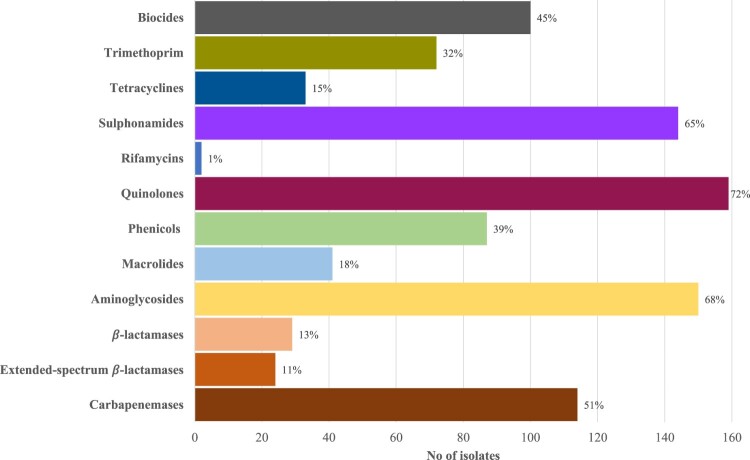
Prevalence of ARGs by antimicrobial classes. Percentages on the bar represent the proportion of isolates which harboured at least one ARG mediating resistance to that antimicrobial class.

Based on the WGS analyses, all isolates harboured the chromosomally-encoded *bla*_OXA-50_, *fosA*, *aph(3’)-IIb*, and *catB7* genes. Excluding these genes, the prevalence of acquired resistance genes (ARGs) from the various drug classes are shown in Figure 1 and Table S2. Most isolates (191/222, 86.0%) harboured at least one ARG from 1 of the 12 classes assessed (median number of classes: 5; range: 0–11). ARGs from the quinolone (159/222, 71.6%) aminoglycoside (150/222, 67.6%), and sulphonamide (144/222, 64.9%) classes were most commonly observed. We did not observe any of the plasmid polymyxin-resistance mediating *mcr* genes.

CNPA harboured a median of six ARGs (range: 0–20) and the most common ARG observed was *crp*P, which encodes transferable ciprofloxacin resistance [28]. Among the predominant STs, isolates from ST308 (Median ARGs: 13, range: 11–13) and ST357 (Median ARGs: 10, range: 0–16) tended to carry more resistance genes.

The prevalence of horizontally acquired carbapenemase genes in our CNPA isolates were high (114/222, 51.4%). Class B metallo-*β*-lactamases (101/222, 45.5%) were predominant – *bla*_IMP_ (47/222, 21.2%), *bla*_NDM-1_ (41/229, 18.5%), and *bla*_VIM_ (13/222, 5.9%). The remaining carbapenemase-producing isolates harboured *bla*_GES-5_ (12/222, 5.4%) and *bla*_KPC-2_ (1/222, 0.5%). There were several variants of *bla*_IMP_ (IMP-1, IMP-7, IMP-13, IMP-43) and *bla*_VIM_ (VIM-2 and VIM-6) observed.

Only two plasmids replicons were identified by PlasmidFinder in our 222 isolates – a IncP-6 replicon in PA0419 (ST3440 KPC-2-producer) and PA0635 (ST244 non-carbapenemase producer), respectively. We manually checked the possible plasmid clusters identified by PlasmidSeeker and found that the contig carrying *bla*_KPC-2_ in PA0419 was similar to the pGSH8-2 plasmid (99.95% identity, 100% coverage) (Accession: AP019194.1). We did not detect any other ARG on this plasmid. pGSH8-2 was described in a multi-drug resistant *Aeromonas hydrophila* which was recovered from the sewage treatment plant in Japan. This *bla*_KPC-2_-carrying plasmid shared the 39–40 kb IncP-6 plasmid replicon backbone with plasmids in other *Aeromonas* spp. strains isolated from wastewater/sewage plants in the USA and China [29]. IncP-6 replicons have been described as a broad range plasmid, with replication ability in *P. aeruginosa*, *Pseudomonas putida* and *Escherichia coli* [30]. Such plasmids have been found to carry *bla*_KPC_ in *P. aeruginosa* (e.g. p10265-KPC, pCOL-1) and may be mobilized between species [31,32]. While KPC carbapenemases appear to be rare in our *P. aeruginosa*, the identification of a broad-range plasmid in our isolate suggests the possibility of hidden *bla*_KPC-2_ environmental reservoirs (especially aquatic environments) or mobilization from other clinically relevant species such as those from the Enterobacterales family where local *bla*_KPC-2_ prevalence is high [33,34].

Unfortunately, we were unable to identify the plasmid identity of PA0635, which could be attributed to the technical difficulties of plasmid identification with short-read sequencing or limitations in the database. Additionally, plasmids were not identified in our major carbapenemase-harbouring ST235 and ST308 clones. Another local study also established that *bla*_NDM-1_ were found on integrative and conjugative elements (ICE), ICE Tn43716385, rather than on plasmids on ST308 *P. aeruginosa* isolates [13].

#### Mutational resistome

The sequence variations in the curated set of chromosomal genes related to *P. aeruginosa* mutational resistance are shown in Table S3. The analysis of the mutational resistome of the CNPA isolates corroborated the propensity of *P. aeruginosa* to acquire chromosomal mutations. A large number of non-synonymous mutations were observed in the various genes studied and it is plausible that these chromosomal mutations contribute to resistance in addition to the ARGs. However, it is also noted that natural polymorphisms/mutational hotspots exist in certain genes such as *ampC*, *mexT, oprD* which are likely not correlated to resistance phenotypes [35,36]. It appears that the frequency of amino acid alterations was fairly similar between carbapenemase- and non-carbapenemase-producing strains.

Mutational inactivation of genes mediating overproduction of chromosomal AmpC cephalosporinase (*ampD*, *ampR*, *dacB*) is one of the most common mutation-driven *β*-lactam resistance mechanism [37]. Additionally, AmpC mutations leading to structural modification of the enzyme and FtsI (encoding penicillin-binding protein 3) mutations have also been associated with ceftolozane/tazobactam and ceftazidime/avibactam resistance [35,38]. There was a total of 28 different *Pseudomonas*-derived cephalosporinase (PDC) variants observed, of which quite a number were reported to be wild-type or have been observed in susceptible strains [35]. The prevalence of deleterious alterations other than AmpC occurred in approximately 50% of the isolates, of which most were not previously reported. However, previously described noteworthy mutations such as AmpR D135N and FtsI F533L, R504C were observed in a few isolates (< 10%).

Efflux pump regulators were frequently mutated, the most common being alterations in ArmZ (143/222, 64.4%), the anti-repressor regulating MexZ repression on MexXY pumps. NalD alterations were also common (99/222, 44.6%), with many isolates harbouring frameshift mutations or premature stop codons. NalD encodes the second repressor that regulates MexAB-OprM expression which is often implicated in carbapenem resistance (except for imipenem) and multi-drug resistance.

Aside from carbapenemase production and efflux mechanisms, porin loss/downregulation due to OprD inactivation is a major contributor in mediating carbapenem resistance. Disrupted porins due to frameshift mutations/premature stop codons/insertion sequences accounted for 128 (57.7%) isolates. The remaining isolates could be largely grouped into five groups where the isolates had high sequence similarity (> 95%) to OprD sequences belonging to wild-type PAO1; clinical strains PA14 (GenBank accession no. ABJ10119), LESB58 (Genbank accession no. YP_002441940), PA-VAP-4 (GenBank accession no. AAS18314.2), and environmental strain MTB-1 (GenBank accession no. CP006853). The distribution of the OprD types is shown in Table 2 Most of these OprD sequences have been observed in carbapenem-susceptible strains, although the shortening of Loop 7 in the LESB58, PA-VAP-4, and MTB-1 variants has been associated with the decreased expression of OprD upon carbapenem exposure [39]. Furthermore, additional amino acid substitutions in these known variants were also observed in some isolates and the impact of these alterations on carbapenem resistance remains to be validated.

**Table 2.** Sub-types of 94 isolates with non-disrupted full-length OprD.

OprD type	No of isolates, *n*	
	100% Identity	Amino acid substitutions
PAO1	6	7
PA14	44	2
LESB58	16	4
MTB-1	4	4
PA-VAP-4	7	0

Mutations in the quinolone-resistance-determining regions QRDRs were also common. Specifically, the T83I GyrA mutation, which has been associated with ciprofloxacin resistance, was detected in 121/222 (54.5%) isolates, frequently accompanied with the S87L ParC mutation (105/122, 86.1%). Moreover, such mutations were found in nearly all ST179, ST308, and ST357 isolates, suggesting an association with particular sequence types.

Finally, mutations related to polymyxin/colistin, aminoglycosides, and fosfomycin appeared less frequently, which might explain the retention of activity in these classes of antibiotics.

## *In silico* MLST and phylogenetic analyses

*In silico* MLST grouped the 222 CNPA isolates into 69 unique STs, 11 of which were novel (Table 3). Out of these, 47 (21.1%) were singletons (represented by one genome). ST235 was the dominant ST (63, 28.5%), followed by ST308 (39, 17.4%), ST244 (12, 5.4%), ST179 (10, 4.5%), and ST357 (10, 4.5%). Together, these five clusters accounted for nearly 60% of the isolates. We observed the occurrence of seven of the world’s top 10 *P. aeruginosa* high-risk clones in our population – ST111, 233, 235, 244, 298, 357 and 308 [40].

**Table 3.** Distribution of STs among non-carbapenase producing and carbapenemase-producing CNPA.

ST	Non-carbapenemase producer (*n *=* *108)	Carbapenemase producer (*n *=* *114)				
		IMP	NDM	VIM	GES-5	KPC
179	10					
**235**	5	39		7	11	
**244**	10			1	1	
**274**	7					
**308**	1		40			
**357**	6	2		2		
Other STs	11(1); 27(2); 111(2); 115(1); 207(1); 245(1); 252(2); 266(1); 282(1); 292(2); 298(1); 313(2); 314(1); **316**(1); 389(1); 399(1); 446(2); 463(1); 471(1); 485(2); 534(1); 553(1); 560(1); 564(1); 569(1); 606(2); 620(1); 664(3); 697(1); 708(1); 792(1); 815(2); 840(1); 882(1); 1076(2); 1247(1); 1342(1); 1666(1); 1930(2); 2013(1); 2021(1); 2033(1); 2069(1); 2326(1); 2476(1); 2651(1); 2651(1); Novel(10)	233(1); 497(1); 621(2); 964(1)	**316**(1)	773(1); 823(2)		Novel(1)

Bold text denotes that ST was observed in both carbapenemase and non-carbapenemase producers.

Carbapenemase-producing CNPA were limited to 12 STs. The most prevalent ST for carbapenemase producers were ST235 and ST308 which accounted for 84.5% of all carbapenemase producers. On the other hand, non-carbapenemase-producing CNPA appeared to be more diverse, being distributed over 63 STs, of which 45 were singletons. The predominant STs were ST179 (*n *=* *10), ST244 (*n *=* *10), and ST274 (*n *=* *7), of which ST179 and ST244 were found solely in non-carbapenamase-producing CNPA. The distribution of carbapenemase, particularly *bla*_NDM_ and *bla*_IMP,_ appear to be related to the ST – *bla*_NDM-1_ were primarily only identified in ST308, while 40 of 47 (85.1%) *bla*_IMP_ isolates were recovered in ST235 isolates.

**Figure 2.**
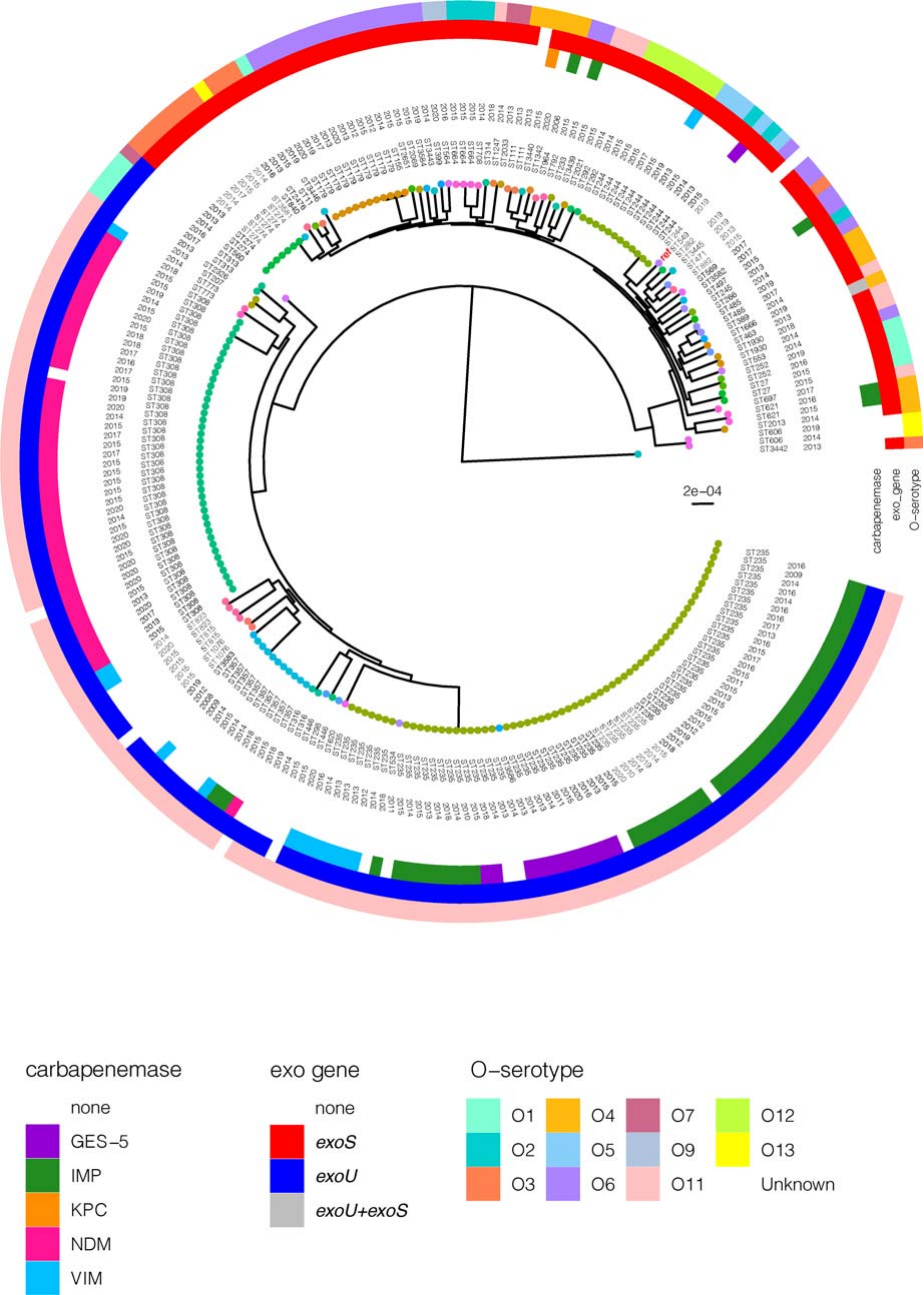
Rooted phylogenetic tree of the 222 *P. aeruginosa* strains in this study inferred from an alignment of 22,810 SNP positions obtained after mapping the genomes to the PAO1 reference genome (highlighted in red and indicated with “ref”) and masking of recombination sites. Isolates are labelled with the sequence types (ST) and year of isolation. The tree tips are coloured by the STs. The colour-coded rings denote the carbapenemase, exo genes, and O-serotype. The scale bar represents the number of nucleotide substitution per site.

WGS analyses identified 22810 high-quality SNPs with reference to *P. aeruginosa* PAO1. There were between 0 and 6670 SNPs (average: 2581) identified between any two isolates. In spite of the diverse STs observed, we noted that there were two main clusters – cluster 1 encompassed the *exo*S+ isolates of varying O-serotypes and typically not carrying any carbapenemase; and cluster 2 comprised the *exo*U+ O-serotype 11 isolates which were primarily carbapenemase-producing (Figure 2). One isolate (PA0817) appeared to be fairly distant from the two main clusters. There was more diversity observed among cluster 1, whereas cluster 2 was represented by a few main clones.

## Discussion

This is the first comprehensive genomic survey of CNPA in Singapore which investigated isolates obtained from the largest public health hospital in the nation. Here, we observed that CNPA with diverse STs which frequently harboured multiple resistance elements, including ARGs and chromosomal mutations, were circulating locally. WGS analyses provided an insight into the molecular epidemiology of local CNPA, including the prevalence of various ARGs and chromosomal mutations, as well as the presence of high-risk epidemic clones.

The population structure of the local CNPA is suggestive of the general population structure of *P. aeruginosa* as described in previous studies, i.e*.* a panmictic non-clonal epidemic structure [41]. Local CNPA isolates are selected from a diverse pool of rare, unrelated genotypes that recombine at high frequency, although selection is biased towards a limited number of widespread clones that represent more than 50% of the sampling population. Similar to previous studies, our CNPA strains were segregated into two large clades, which is characterized by the presence of *exo*S and *exo*U, although we did not find any distinguishing clinical features between the two clades [42].

The most prevalent clones observed in our study were isolates from the ST235 and ST308 clades, both of which were primarily carbapenemase-producing. Given the geographical position of Singapore as an international travel and medical hub, the observation of ST235 is not surprising as this is a highly prevalent, widely-disseminated global clone which has been reported in Europe, Asia, America. It has also been associated with outbreaks within specific countries [43]. Similar to previous reports, we note a diversity in the resistance determinants, particularly the carbapenemases, among the local ST235 isolates. IMP variants, VIM-2, and GES-5 were distributed in the isolates over the survey years from 2009 to 2020. This suggests independent acquisition events over the survey period as opposed to a clonal dissemination/expansion, which is in line with previous observations [44,45]. Additionally, we note that this is the first description of GES-5-harbouring *P. aeruginosa* in Singapore, where the first isolate was observed in 2011. Much like the GES-5-harbouring *P. aeruginosa* strains identified in the region (Japan, Indonesia), it appears that GES-5 in this study was associated with the ST235 clone; GES-5 was only identified in ST235 except one isolate (ST244) [46,47].

ST308, the other dominant clone in our population, appeared to be much more genetically similar compared to ST235 (ST308 core SNPs: 21, range: 0–6; ST235 core SNPs: 78, range 0–26), displaying uniform resistance phenotypes and genotypes. All except one isolate were NDM-1-harbouring and most isolates possessed the same set of acquired and mutational resistome. This might suggest a clonal expansion, which is also indicated by the low SNP differences. Importantly, this clone appears to harbour a high number of ARGs and chromosomal mutations.

The first observation of ST308 in our survey was in 2013, which coincided with the increasing prevalence of carbapenem-resistant Enterobacterales in Singapore [34]. As the major clone which carried the NDM carbapenemase, the introduction of this clone contributed largely to the increase in the diversity of carbapenemases within the institution. Historically, IMP variants were the primary metallo-*β*-lactamase described in the local population, with sporadic detection of VIM carbapenemases [11,12]. While IMP variants continue to be the predominant carbapenemase in our survey, we noted a relatively higher proportion of NDM-producers in recent years, contributed by the uptick in ST308 clones. This is in contrast to the epidemiology of metallo-*β*-lactamase-producing CNPA, where NDM production typically tends to be rare [48,49]. Furthermore, our findings appear to be congruent to previous studies which suggest that ST308 *P. aeruginosa*, specifically those carrying NDM-1, appear to be circulating in the Southeast Asian region. Elsewhere, ST308 resistant *P. aeruginosa* isolates are more commonly associated with VIM or IMP [40]. In another local acute care public hospital, an ongoing persistent spread of ST308 NDM-1-producing *P. aeruginosa* has been reported [13]. Likewise, ST308 NDM-1-producing *P. aeruginosa*, with 100% homology of the *bla*_NDM_ nucleotide sequence with the gene isolated from a Singapore strain, has been observed in Malaysia [50].

Although the phylogenetic findings appear to suggest a clonal transmission of ST308 CNPA, the clinico-epidemiological findings are not indicative of a single outbreak occurrence as the strains were isolated through the years dating from 2013 to 2020 and were not confined to a single ward. We postulate that there is likely an unknown persistent environmental reservoir sustaining the continued dissemination and transmission of the clone within the hospital environment. Comparative genomic studies performed for ST308 outbreaks have demonstrated high relatedness between clinical and environmental strains, particularly those from the hospitals’ wastewater systems and washing basins [13,51–53]. Continued surveillance of ST308 *P. aeruginosa* is critical, as previous studies have demonstrated the difficulties in eradicating *P. aeruginosa* in the hospital environment in spite of stringent infection control and cleaning measures, leading to sustained outbreaks [52,54]. In fact, our institution reported a polymyxin-susceptible only ST357 VEB-1-harbouring *P. aeruginosa* outbreak in 2009 where the source was traced to the sinks’ drainage system in the haematological wards [55,56]. Fortunately, we observed only isolated cases of ST357 *P. aeruginosa* in this survey.

Majority of our CNPA isolates exhibited broad-spectrum resistance, with a high rate of MDR/DTR phenotypes, leaving virtually no therapeutic alternatives with the exception of the highly toxic polymyxins. CNPA also often harboured many ARGs from various antimicrobial classes, including biocides. Carbapenemase production is a major contributor to carbapenem resistance in the CNPA strains in Singapore, accounting for more than 50% of all isolates in this study. This is in contrast to other epidemiological studies conducted in the United States and Europe, where much lower carbapenemase rates (4–28%) were observed in carbapenem-resistant or MDR strains [57–59]. The prevalence rate observed here is more similar to those in other Asian countries [60]. Carbapenem resistance in the non-carbapenemase-producing isolates can largely be explained by a combination of AmpC hyperproduction, porin deficiency, and efflux mechanisms. Importantly, these mechanisms also exist in carbapenemase-producing strains, which should be taken into consideration when designing treatment strategies, particularly in the choice of combination therapy. Additionally, plasmids could play a role in mediating ARG transfers such as carbapenemases which are infrequently found in *P. aeruginosa.* It may be worth investigating other ARG-harbouring isolates recovered from hospital patients and the environment to determine the presence of plasmid reservoirs which may be responsible for ongoing transmission.

Our study is limited in its retrospective nature and involved only a single centre. We are unable to determine the full prevalence data as not all CNPA isolates detected during the study period were available/sequenced. Moreover, we did not perform any gene expression studies which could provide additional insights into carbapenem resistance and relevance to the chromosomal mutations observed. However, we believe that our findings are largely congruent to other studies, for instance the detection of ST308 in another local hospital [13]. Additionally, the lack of clinical epidemiological information precluded our ability to identify outbreaks.

In conclusion, this study demonstrates the use of WGS to provide comprehensive epidemiological information on CNPA isolates from a large acute care hospital in Singapore, which remained scarce locally and in the Southeast Asian region. Attention should be paid to the high carbapenemase prevalence and emerging high-risk clones detected, suggesting a greater emphasis on infection control measures. Molecular surveillance of CNPA is of utmost importance, especially since *P. aeruginosa* is ubiquitous and can reside in the environment. Genomic data together with clinical epidemiological information can provide valuable guidance to infection control and prevention strategies. To do so, continuous surveillance programmes integrating WGS and traditional clinic-epidemiological methods are desirable. This study provides a snapshot of the CNPA problem in Singapore and serves as valuable baseline information to benchmark against future surveillance studies.

## Supplementary Material

tables_supplementary.xlsx
